# Switching From Daily DPP-4 Inhibitor to Once-Weekly GLP-1 Receptor Activator Dulaglutide Significantly Ameliorates Glycemic Control in Subjects With Poorly Controlled Type 2 Diabetes Mellitus: A Retrospective Observational Study

**DOI:** 10.3389/fendo.2021.714447

**Published:** 2021-08-06

**Authors:** Junpei Sanada, Tomohiko Kimura, Masashi Shimoda, Akiko Tomita, Yoshiro Fushimi, Tomoe Kinoshita, Atsushi Obata, Seizo Okauchi, Hidenori Hirukawa, Kenji Kohara, Fuminori Tatsumi, Shuhei Nakanishi, Tomoatsu Mune, Kohei Kaku, Hideaki Kaneto

**Affiliations:** Department of Diabetes, Endocrinology and Metabolism, Kawasaki Medical School, Kurashiki, Japan

**Keywords:** DPP-4 inhibitor, weekly GLP-1 receptor activator, dulaglutide, glycemic control, Hba1c, propensity score matching

## Abstract

**Aim:**

At present, daily DPP-4 inhibitors are quite frequently prescribed in subjects with type 2 diabetes mellitus (T2DM). Recently, it has been drawing much attention that once-weekly incretin-based injection dulaglutide was developed. In this study, we aimed to examine the possible effects of once-weekly GLP-1 receptor activator (GLP-1RA) dulaglutide on glycemic control as well as various metabolic parameters.

**Methods:**

We made a direct comparison between the effect of daily DPP-4 inhibitor and once-weekly dulaglutide on glycemic control in “study 1 (pre–post comparison)” and set the control group using the propensity score matching method in “study 2”.

**Results:**

In study 1, switching from daily DPP-4 inhibitor to dulaglutide significantly ameliorated glycemic control in subjects with T2DM. Such effects were more obvious in poorly controlled subjects. After 1:1 propensity score matching, the switching group improved glycemic control compared with the non-switching group in study 2.

**Conclusion:**

We should bear in mind that switching from daily DPP-4 inhibitor to once-weekly GLP-1RA dulaglutide exerts more favorable effects on glycemic control regardless of age, body weight, and duration of diabetes in subjects with T2DM, especially when we fail to obtain good glycemic control with daily DPP-4 inhibitor.

## Introduction

Type 2 diabetes mellitus (T2DM) is well known to bring about both micro- and macroangiopathy when glycemic control is inadequate. Several large clinical trials including the DCCT, UKPDS, and Kumamoto studies have demonstrated that strict glycemic control prevents the development of microangiopathy in patients with type 1 and type 2 diabetes (1–3). In contrast, the ACCORD and ADVANCE studies failed to show the significance of obtaining strict glycemic control for the prevention of macroangiopathy (4, 5). Furthermore, the ACCORD study showed that strict glycemic control led to an increased number of deaths, which was presumably due to severe hypoglycemia induced by intensive glycemic control. From the perspective of these points, meticulous glycemic control without hypoglycemia is essential for the treatment of diabetes to prevent its complications (6–8).

Recently, incretin-based medicine is commonly prescribed in routine medical care due to their outstanding properties and convenience. Incretin preparations promote insulin secretion depending on blood glucose levels, and thereby the risk of hypoglycemia is very low with monotherapy of such medicine. There are two types of incretin-based medicine such as dipeptidyl peptidase-4 (DPP-4) inhibitor and GLP-1 receptor activator (GLP-1RA). DPP-4 inhibitor, which is easy for non-specialists to handle, is frequently used in daily clinical practice. DPP-4 inhibitor acts by suppressing the enzyme activity that breaks down GLP-1, thus increasing the level of serum GLP-1. However, this is not able to raise serum GLP-1 concentrations up to high levels found after injection of GLP-1RA, and therefore, its glucose-lowering efficacy is less than that of GLP-1RA (9). According to the report of the Ministry of Health, Labor and Welfare in Japan at 2017, DPP-4 inhibitor accounted for over 40% of the total sales and is the most often used drug in clinical practice. Another incretin-based medicine, GLP-1RA, accounted for only 5% of the total sales due to the inconvenience of being an injectable drug. However, considering the mechanism of these drugs, it would be theoretically effective to change from DPP-4 inhibitor to GLP-1RA in subjects who are taking DPP-4 inhibitor but have insufficient blood glucose control. Recently, it was revealed the use of GLP-1RA was related to lower mortality compared with DPP-4 inhibitors or placebo (10). The reason for the low usage rate of GLP-1RAs with such high effectiveness is thought to be due to the inconvenience of being an injectable drug. In recent years, the highly convenient weekly GLP-1RA dulaglutide is available in routine medical care. In this study, we changed DPP-4 inhibitor to dulaglutide in poorly controlled T2DM subjects who were taking DPP-4 inhibitor, and then we examined the changes in various metabolic parameters.

## Methods

### Study Population and Patient Preparation

We performed this study with outpatients retrospectively in our institution from October 1 in 2016 to August 31 in 2017. The study protocol including the Opt-out informed consent was approved by Institutional Review Board of Kawasaki Medical School (No. 2899). The study was conducted in accordance with the Declaration of Helsinki. Since this study was retrospective, instead of obtaining informed consent from each patient, we provided public information about the study *via* the hospital homepage as described below. “For the purpose of investigating the efficacy in changing from DPP-4 inhibitors to dulaglutide, we will retroactively collect clinical data on patients taking DPP-4 inhibitors who visited our department between October 1, 2016 and August 31, 2017. If you disagree with this research, please contact us and we will exclude you. Moreover, there is no disadvantage even if you do not participate in this research.” Namely, patients were included in the research unless they expressly requested to be excluded. Enrolled subjects in this study fulfilled the following criteria: HbA1c ≥ 7.0%, having already taken DPP-4 inhibitor in subjects with T2DM. Additionally, the subjects fulfilled the below-described criteria: (1) without severe renal dysfunction; (2) without severe liver dysfunction; (3) without infectious disorder, malignancy, or various endocrine disorders; and (4) without using steroid preparation. We switched from only DPP-4 inhibitor to 0.75 mg of weekly GLP-1RA dulaglutide. We compared HbA1c, blood glucose, and another metabolic parameter before and 6 months after the switching. We enrolled 37 subjects (male/female = 13/24) in total in this study, and then we collected the data for variables such as clinical parameters and type of medication. Next, for setting the control group, subjects who continued to receive DPP-4 inhibitors without changing their antidiabetic drug during the study period were then extracted and matched to 1:1 using the propensity score with the dulaglutide-switching group. Data from 3 months before switching were also used in this study.

### Statistical Analysis

We used JMP version 13 (SAS Institute Inc., North Carolina, USA) in all statistical analyses. A paired *t*-test was used to compare the differences at time points for the same group, and we used Student’s *t*-test and Mann–Whitney *U*-tests for comparing the difference between the switching group and non-switching group. *χ* ^2^ test was used for comparing the usage rate of concomitant drugs between two groups. In the abovementioned analyses, we regarded *p* < 0.05 as significant. In study 2, to minimize the imbalance in the clinical features between each group, propensity score was defined as the estimated probability of patients to start a switching group *vs*. a non-switching group, estimated from a logistic regression model that included five predefined baseline covariates such as age, sex, duration of diabetes, baseline HbA1c, and baseline BMI. The 1:1 propensity score-matched cohort was created using a 1:1 nearest-neighbor matching algorithm (with maximum caliper width of 0.20). To elucidate what kind of factors are correlated with ΔHbA1c, we performed Spearman’s rank correlation coefficient test. The results were expressed as mean ± SD.

## Results

### Clinical Characteristics in the Present Study Subjects

#### Study 1

In this study, we enrolled 37 subjects (male/female = 13/24) in total (**Table 1A**). The characteristics of the present study subjects at baseline were as described below: age, 56.2 ± 13.6 years old; BMI, 31.7 ± 5.9 kg/m^2^; duration of diabetes, 14.4 ± 7.7 years; HbA1c, 8.7 ± 1.3%; and PG, 176.2 ± 49.4 mg/dl. The frequencies of diabetic neuropathy, retinopathy, and nephropathy (urinary albumin ≥ 30 mg/gCr) were 59.5%, 35.1%, and 54.1%, respectively. The frequencies of ischemic heart disease and stroke were 5.4% and 5.4%, respectively. The percentage of using DPP-4 inhibitor, metformin, thiazolidine, sulfonylurea, glinide, α-glucosidase inhibitor, SGLT2 inhibitor, and insulin preparation was 100%, 78.4%, 40.5%, 32.4%, 10.8%, 13.5%, 48.6%, and 21.6%, respectively. Usage rate of antihypertensive drug and lipid-lowering drug was 51.4% and 78.4%, respectively. There were no dosage changes in any drug for 6 months after switching.

**Table 1:** Clinical baseline characteristics of the subjects in this study.

A. Study 1.					
parameter	mean	± SD	parameter	mean	± SD
Number	37		HDL-C (mg/dL)	47.9	9.9
Gender (M/F)	13/24		LDL-C (mg/dL)	108.5	26.4
Age (years)	56.2	13.6	ɤ-GTP (U/L)	50.5	37.8
Duration (years)	14.4	7.7	ALT (U/L)	39.1	32.0
BMI (kg/m^2^)	31.7	5.9	AST (U/L)	32.5	20.4
HbA1c (%)	8.7	1.3	Cre (mg/dL)	0.8	0.5
PG (mg/dL)	176.2	49.4	BUN (mg/dL)	15.9	5.6
TG (mg/dL)	204.9	114.6	UA (mg/dL)	5.1	1.1
B. Study 2					
parameter	non-switching group		Switching group		*p* value
Age (years)	57.6 ± 11.9		55.1 ± 12.4		n.s.
Gender (M/F)	8/12		7/13		n.s.
Duration (years)	16.0 ± 15.9		15.2 ± 8.4		n.s.
BMI (kg/m^2^)	29.5 ± 6.0		28.9 ± 4.2		n.s.
HbA1c (%)	8.2 ± 1.2		8.2 ± 0.8		n.s.
PG (mg/dL)	188.3 ± 77.9		159.3 ± 45.6		n.s.
TG (mg/dL)	110.9 ± 25.4		120.2 ± 26.9		n.s.
HDL-C (mg/dL)	49.1 ± 13.1		47.4 ± 11.3		n.s.
LDL-C (mg/dL)	103.5 ± 27.8		113.6 ± 30.5		n.s.
ɤ-GTP (U/L)	38.7 ± 39.7		38.7 ± 39.7		n.s.
ALT (U/L)	24.3 ± 12.1		24.3 ± 12.1		n.s.
AST (U/L)	24.2 ± 9.3		24.2 ± 9.3		n.s.
Cre (mg/dL)	0.7 ± 0.2		0.8 ± 0.5		n.s.
BUN (mg/dL)	16.0 ± 4.7		16.0 ± 4.7		n.s.
UA (mg/dL)	4.9 ± 1.0		4.8 ± 0.8		n.s.

BMI, body mass index; PG, plasma glucose; TG, triglyceride; HDL-C, high-density lipoprotein cholesterol; LDL-C, low-density lipoprotein cholesterol; ɤ-GTP, ɤ-glutamyl trans peptidase; ALT, alanine aminotransferase; AST, aspartate aminotransferase, Cre, creatinine; BUN, blood urea nitrogen; UA, uric acid. Data are shown mean ± SD.

n.s., not significant.

#### Study 2 (Propensity Score Matching)

Next, we set the control group who were continuously receiving DPP-4 inhibitor without any change in anti-diabetic drugs during the same period in this study. The number of patients was 460. Secondly, to minimize the possible imbalance in the clinical parameters between each group at the baseline, we used 1:1 propensity score matching including five predefined baseline covariates: age, gender, duration of diabetes, HbA1c, and BMI. Finally, each of the 20 cases was matched 1:1. The clinical characteristic of baseline is shown in **Table 1B**. The percentage of concomitant drugs in the non-switching group and switching group was as follows: DPP-4 inhibitor (100% *vs*. 100%), metformin (65.0% *vs*. 85.0%), thiazolidine (45.0% *vs*. 45.0%), sulfonylurea (55.0% *vs*. 30.0%), glinide (25.0% *vs*. 15.0%), α-glucosidase inhibitor (10.0% *vs*. 5.0%), SGLT2 inhibitor (20.0% *vs*. 40.0%), and insulin preparation (10% *vs*. 20%). There were no significant differences in the usage of concomitant drugs between two groups.

### Evaluation of Glucose and Lipid Parameters After Switching From DPP-4 Inhibitor to Weekly GLP-1RA Dulaglutide

#### Study 1

**Table 2A** shows the parameters from baseline to 3 months and 6 months later. BMI significantly decreased after 3 months compared to baseline (from 31.7 ± 5.9 to 30.5 ± 5.1 kg/m^2^). However, this change disappeared 6 months after switching. Glycemic control and HbA1c were significantly ameliorated after 3 months and 6 months (from 8.7% ± 1.3% to 7.9% ± 1.0% and 7.9% ± 1.1%). There was no change in plasma glucose (PG) from baseline to 3 months later, but PG was significantly improved 6 months later (from 176.2 ± 49.4 to 157.2 ± 47.3 and 155.1 ± 43.3 mg/dl). Lipid profile, such as TG, HDL-C, and LDL-C, did not change from baseline to until 6 months later (TG: from 204.9 ± 114.6 to 186.0 ± 95.1 mg/dl, HDL-C: from 47.9 ± 9.9 to 48.4 ± 10.0 mg/dl, LDL-C: from 108.5 ± 26.4 to 108.1 ± 29.7 mg/dl). Hepatic enzymes (ALT, AST, and γGTP) also did not change from baseline to until 6 months later (ALT: from 39.1 ± 32.0 to 37.3 ± 27.5 U/L, AST: from 32.5 ± 20.4 to 31.1 ± 17.0 U/L, γGTP: from 50.5 ± 37.8 to 54.0 ± 49.5 U/L). Serum creatinine and BUN were normal levels at baseline. BUN level was elevated 6 months later compared to baseline; however, it remained within the normal reference range (from 15.9 ± 5.6 to 17.3 ± 6.7 mg/dl). Creatinine levels did not change until 6 months later (from 0.8 ± 0.5 to 0.8 ± 0.5 mg/dl). We evaluated the relationship between ΔHbA1c (6 months − baseline) and various clinical parameters at baseline (**Table 3A**). Age, duration, BMI, ΔBMI (6 months − baseline), and lipid parameter were not related to ΔHbA1c for 6 months. There was a trend between ΔBMI and ΔHbA1c even though it did not reach statistical significance (*ρ* = 0.3154, *p* = 0.0609). The higher the HbA1c and PG levels, the greater effect in the improvement of ΔHbA1c after 6 months (*ρ* = −0.3485 and −0.279, *p* = 0.0345 and 0.0008, respectively).

**Table 2:** Time course of various clinical parameters after switching from DPP-4 inhibitor to dulaglutide.

A. Study 1								
parameter	baseline			3 months			6 months	
BMI (kg/m^2^)	31.7 ± 5.9			30.5 ± 5.1*			31.4 ± 6.0	
HbA1c (%)	8.7 ± 1.3			7.9 ± 1.0*			7.9 ± 1.1*	
PG (mg/dL)	176.2 ± 49.4			157.2 ± 47.3			155.1 ± 43.3*	
TG (mg/dL)	204.9 ± 114.6			189.4 ± 135.6			186.0 ± 95.1	
HDL-C (mg/dL)	47.9 ± 9.9			48.1 ± 10.6			48.4 ± 10.0	
LDL-C (mg/dL)	108.5 ± 26.4			107.1 ± 33.1			108.1 ± 29.7	
γ-GTP (U/L)	50.5 ± 37.8			52.1 ± 42.1			54.0 ± 49.5	
ALT (U/L)	39.1 ± 32.0			38.4 ± 29.3			37.3 ± 27.5	
AST (U/L)	32.5 ± 20.4			30.2 ± 15.8			31.1 ± 17.0	
Cre (mg/dL)	0.80 ± 0.5			0.8 ± 0.4			0.8 ± 0.5	
BUN (mg/dL)	15.9 ± 5.6			15.9 ± 5.6			17.3 ± 6.7*	
UA (mg/dL)	5.1 ± 1.1			5.2 ± 1.2			5.3 ± 1.2	
B. Study 2								
Parameter	non-switching group				switching group			
	**-3 months**	**baseline**	**3 months**	**6 months**	**-3 months**	**baseline**	**3 months**	**6 months**
BMI (kg/m^2^)	30.0 ± 6.0	29.5 ± 6.0	29.7 ± 6.0	29.9 ± 6.1	28.6 ± 4.3	28.9 ± 4.2	28.3 ± 4.2	28.9 ± 4.6
HbA1c (%)	8.0 ± 1.2	8.2 ± 1.2	7.9 ± 0.9	8.1 ± 1.2	8.1 ± 1.1	8.2 ± 0.8	7.6 ± 0.7*	7.6 ± 0.9*
PG (mg/dL)	184.5 ± 69.4	188.3 ± 77.9	167.4 ± 49.7	185.0 ± 47.9	166.3 ± 67.8	159.3 ± 45.6	159.9 ± 49.5	143.2 ± 41.2^§^
TG (mg/dL)	193.0 ± 167.2	184.0 ± 110.9	180.5 ± 158.4	178.3 ± 119.1	193.1 ± 89.2	217.2 ± 120.2	170.6 ± 60.2	181.7 ± 66.0
HDL-C (mg/dL)	47.5 ± 11.9	49.1 ± 13.1	50.0 ± 14.0	51.7 ± 11.2	47.7 ± 13.0	47.4 ± 11.3	47.0 ± 11.1	47.8 ± 10.6
LDL-C (mg/dL)	95.9 ± 22.8	103.5 ± 27.8	100.3 ± 24.1	107.0 ± 26.4	111.9 ± 33.4	113.6 ± 30.5	103.2 ± 30.2	104.1 ± 25.1
γ-GTP (U/L)	34.1 ± 33.3	38.7 ± 39.7	42.1 ± 51.5	38.1 ± 42.3	41.2 ± 37.3	39.4 ± 31.5	39.4 ± 31.5	38.4 ± 34.9
ALT (U/L)	23.2 ± 10.5	24.3 ± 12.1	25.1 ± 17.0	24.6 ± 12.2	40.0 ± 30.6	41.5 ± 35.7	38.9 ± 30.7	39.2 ± 29.2
AST (U/L)	23.3 ± 8.8	24.2 ± 9.3	23.6 ± 10.6	24.5 ± 8.8	30.4 ± 18.4	34.7 ± 24.4	29.0 ± 15.1	31.9 ± 20.1
Cre (mg/dL)	0.7 ± 0.2	0.7 ± 0.2	0.7 ± 0.1	0.7 ± 0.1	0.8 ± 0.4	0.8 ± 0.5	0.8 ± 0.4	0.8 ± 0.5
BUN (mg/dL)	16.5 ± 4.1	16.0 ± 4.7	16.6 ± 4.5	16.6 ± 4.6	16.4 ± 7.0	15.6 ± 5.6	15.4 ± 5.8	17.0 ± 6.7*
UA (mg/dL)	5.2 ± 1.0	4.9 ± 1.0	5.1 ± 1.0	4.8 ± 1.0	5.1 ± 1.0	4.8 ± 0.8	4.9 ± 1.1	5.0 ± 1.1

BMI, body mass index; PG, plasma glucose; TG, triglyceride; HDL-C, high-density lipoprotein cholesterol; LDL-C, low-density lipoprotein cholesterol; γ-GTP, γ-glutamyl trans peptidase; ALT, alanine aminotransferase; AST, aspartate aminotransferase, Cre, creatinine; BUN, blood urea nitrogen; UA, uric acid; n.s., not significant. Data are shown mean ± SD. *p < 0.05 *vs* baseline. ^§^p < 0.05 *vs* non-switching at each time point.

**Table 3:** Univariate analysis evaluating the association between Δ HbA1c (6 months - baseline) and various clinical parameters at baseline.

A. Study 1		
Univariate analysis		
	△HbA1c	
parameter	ρ	*p*
Age (years)	-0.0895	n.s.
Duration (years)	0.0621	n.s.
BMI (kg/m^2^)	0.1786	n.s.
△BMI (kg/m^2^)	0.3154	0.0609
HbA1c (%)	-0.3485	0.0345
PG (mg/dL)	-0.5279	0.0008
TG (mg/dL)	-0.0928	n.s.
HDL-C (mg/dL)	-0.0955	n.s.
LDL-C (mg/dL)	-0.1500	n.s.
B. Study 2		
Univariate analysis (switching)		
	△HbA1c	
parameter	ρ	*p*
Age (years)	0.1048	n.s.
Duration (years)	0.1105	n.s.
BMI (kg/m^2^)	0.1730	n.s.
△BMI (kg/m^2^)	0.4711	0.0417
HbA1c (%)	-0.2908	n.s.
PG (mg/dL)	-0.4535	0.0446
TG (mg/dL)	-0.2899	n.s.
HDL-C (mg/dL)	-0.1376	n.s.
LDL-C (mg/dL)	-0.4342	n.s.

BMI, body mass index; △BMI, (BMI at 6 months – BMI at baseline); PG, plasma glucose; TG, triglyceride; HDL-C, high-density lipoprotein cholesterol; LDL-C, low-density lipoprotein cholesterol. n.s., not significant.

### Comparison of Other Clinical Parameters Between the Switching Group From DPP-4 Inhibitor to Weekly GLP-1RA Dulaglutide and the Non-Switching Group

#### Study 2 (Propensity Score Matching)

At baseline, all parameters showed the same level between the two groups (**Table 1B**). As shown in **Table 2B**, there was no significant difference in each value between baseline and before 3 months from baseline in each group. This suggested that the reason for the switching was not due to the acute deterioration of glycemic control. In the non-switching group, there were no significant change in all parameters compared to that of baseline. In contrast, in the switching group, HbA1c was improved both 3 months and 6 months after switching from DPP-4 inhibitors although there was no significant change in BMI. PG at 6 months later was lower in the switching group compared to the non-switching group. There were no changes in lipid profile (TG, HDL-C, and LDL-C) and liver enzymes (ALT and AST). BUN level was slightly elevated from baseline (15.6 ± 5.6 mg/dl) to 6 months after switching (17.0 ± 6.7 mg/dl) even though it was still in the normal range. We compared the changes for 6 months between the non-switching group and the switching group (**Table 4**). There was a significant difference in changes in HbA1c and BMI (*p* = 0.006 and 0.026, respectively). We performed Spearman’s rank correlation coefficient test for evaluating the relationship between ΔHbA1c (6 months − baseline) and baseline parameters (**Table 3B**). The positive correlation between ΔBMI and ΔHbA1c was revealed (*ρ* = 0.4711, *p* = 0.0417). Furthermore, PG negatively associated with ΔHbA1c (ρ= −0.4535, *p* = 0.0446).

**Table 4:** Comparison of the changes of various parameters for 6 months between non-switching group and switching group. Study 2.

parameter	non-switching group	switching group	*p* value
Δ HbA1c (%)	0.005 ± 1.214	-0.625 ± 0.836	0.006
Δ BMI (kg/m^2^)	0.335 ± 0.744	-0.338 ± 1.122	0.026
Δ PG (mg/dL)	-8.5 ± 80.4	-16.1 ± 42.0	n.s.

BMI, body mass index; PG, plasma glucose; n.s., not significant. Data are shown mean ± SD.

## Discussion

In this study, we switched from DPP-4 inhibitors to weekly GLP-1RA dulaglutide in subjects with T2DM. Six months after the switching, glycemic control was significantly improved from the baseline whereas there were no changes in body weight as well as other metabolic parameters. The higher HbA1c at switching, the greater decrement was seen in HbA1c after 6 months. While it was thought that GLP-1RA exerted stronger effects on glycemic control compared with DPP-4 inhibitors (9, 11), our data in this study clearly showed the superiority of dulaglutide on glycemic control compared to DPP-4 inhibitors. In particular, it was more effective in patients with poor blood glycemic control with DPP-4 inhibitors. In addition, this effect could be confirmed by setting and analyzing controls by propensity score matching. This is reasonable considering the molecular mechanism of its action. In other words, GLP-1RA therapy brings large amounts of exogenous ligands and thus it is more effective than DPP-4 inhibitor that maintains a serum active GLP-1 level. On the other hand, there was no statistical difference in body weight loss after the switching. One possible reason for this is thought to be that long-acting formulations such as dulaglutide have a weaker effect of delaying gastric emptying and have less gastrointestinal symptoms than short-acting formulations (12). The relationship between ΔBMI and ΔHbA1c was interesting; there was no significant statistical difference in study 1. On the other hand, the positive correlation was observed in study 2. Generally, it is considered that dulaglutide 0.75 mg itself has little weight-reduction effect. However, it is considered that glycemic control was improved as body weight was reduced, although it is unclear whether this correlation was the direct effect of this drug. There is a report regarding the effectiveness between sitagliptin and liraglutide. Switching from sitagliptin to liraglutide significantly decreased the HbA1c level as well as body weight reduction. The authors analyzed that it was due to increased gastrointestinal reaction (13). However, in real clinical practice, we think that this is the first report comparing the effect between DPP-4 inhibitor and GLP-1RA dulaglutide after switching.

Recently, it has been established that GLP-1RA has an anti-arteriosclerotic effect and reduces cardiovascular events in high-risk type 2 diabetic patients (14–17). In fact, with the revision of the ADA/EASD consensus guidelines published in 2018 (18), much attention has been focused on GLP-1RA. GLP-1RA is defined as a second-line drug as well as a sodium glucose co-transporter (SGLT) 2 inhibitor in subjects with established arteriosclerotic disease or chronic kidney disease. GLP-1RA not only has a blood glucose-lowering effect but also has an anti-arteriosclerotic effect and protective effect against chronic kidney disease (15). The effect of anti-arteriosclerosis is considered to be not only from the improvement of metabolic parameters but also from direct effect through the GLP-1 receptor expressed in vascular endothelial cells (19–21). Moreover, considering the molecular mechanism of GLP-1RA against β-cell failure, earlier usage of GLP-1RA is extremely important. Indeed, we reported that both DPP-4 inhibitor and GLP-1RA had a protective effect against β-cell dysfunction in a diabetic rodent model (22–24) and that the effectiveness was more remarkable at the early phase of diabetes compared with the advanced stage of diabetes (24). As a matter of course, meticulous glycemic control without hypoglycemia is very important for the management of T2DM. Therefore, we assume that GLP-1RA would be promising from this point of view, although, needless to say, further prospective study with larger number of subjects is necessary to conclude this point. In addition, it is known that, in general, when some receptor is chronically exposed to large amounts of its ligand, such receptor expression is downregulated (25). Therefore, it has been concerning that the long-term usage of GLP-1RA would downregulate its receptor expression, because it is known that serum GLP-1 level increases up to non-physiological concentrations after GLP-1RA treatment. We reported, however, that long-term usage of GLP-1RA dulaglutide did not attenuate the GLP-1 receptor expression level in pancreatic β-cells and thereby contributed to maintaining good glycemic control (26). Recently, the term “clinical inertia” has been attracting much attention following the publication of ADA/EASD consensus guidelines 2018 (18). Not only patients but also medical staff tend to hesitate to change to injectable treatment. However, these results indicate the possibility that switching from DPP-4 inhibitor to dulaglutide leads to improved glycemic control in subjects with poor glycemic control.

There are some limitations in this study. First, the number of subjects was small especially in study 2, because the whole patient number of this study was relatively small (*n* = 37) and then we used a 1:1 propensity matching method. However, we believe that this background factor-adjusted analysis produces more objective data in such retrospective study. Second, we were unable to obtain sufficient C-peptide or insulin data to withstand the analysis due to the characteristic of this kind of clinical retrospective study. It is very interesting how switching from DPP-4 inhibitor to dulaglutide affects the improvement of glycemic control, depending on residual pancreatic β-cell function. Further prospective clinical trials would be necessary to evaluate the efficacy of dulaglutide after switching from DPP-4 inhibitor.

In conclusion, changing from DPP-4 inhibitors to dulaglutide was more effective in glycemic control in subjects with T2DM. These data suggest that step-up treatment from DPP-4 inhibitor to GLP-1RA is very promising especially in cases with insufficient glycemic control.

## Data Availability Statement

The raw data supporting the conclusions of this article will be made available by the authors, without undue reservation.

## Ethics Statement

The studies involving human participants were reviewed and approved by Institutional Review Board of Kawasaki Medical School. Written informed consent for participation was not required for this study in accordance with the national legislation and the institutional requirements.

## Author Contributions

JS, TKim, and HK designed research. JS, TKi, MS, AT, YF, TKin, AO, SO, HH, KKo, and FT contributed to data collection. All authors treated patients and reviewed the manuscript. JS and TKim analyzed the data and wrote the manuscript. KKa advised interpretation of data. HK reviewed and edited the manuscript. All authors contributed to the article and approved the submitted version.

## Conflict of Interest

KKa has been an advisor to, received honoraria for lectures from, and received scholarship grants from Novo Nordisk Pharma, Sanwa Kagaku Kenkyusho, Takeda, Taisho Pharma, MSD, Kowa, Sumitomo Dainippon Pharma, Novartis, Mitsubishi Tanabe Pharma, AstraZeneca, Boehringer Ingelheim, Chugai, Daiichi Sankyo, and Sanofi. HK has received honoraria for lectures, received scholarship grants, and received research grant from Novo Nordisk Pharma, Sanofi, Eli Lilly, Boehringer Ingelheim, Taisho Pharma, Sumitomo Dainippon Pharma, Takeda Pharma, Ono Pharma, Daiichi Sankyo, Mitsubishi Tanabe Pharma, Kissei Pharma, MSD, AstraZeneca, Astellas, Novartis, and Kowa. However, this study was not funded by the above funders.

The remaining authors declare that the research was conducted in the absence of any commercial or financial relationships that could be construed as a potential conflict of interest.

## Publisher’s Note

All claims expressed in this article are solely those of the authors and do not necessarily represent those of their affiliated organizations, or those of the publisher, the editors and the reviewers. Any product that may be evaluated in this article, or claim that may be made by its manufacturer, is not guaranteed or endorsed by the publisher.

## Abbreviations

BMI, body mass index; PG, plasma glucose; TG, triglyceride; HDL-C, high-density lipoprotein cholesterol; LDL-C, low-density lipoprotein cholesterol; ɤ-GTP, ɤ-glutamyl trans peptidase; ALT, alanine aminotransferase; AST, aspartate aminotransferase; Cre, creatinine; BUN, blood urea nitrogen; UA, uric acid.
